# Upregulation of vitamin D-binding protein is associated with changes in insulin production in pancreatic beta-cells exposed to *p*,*p*′-DDT and *p,p*′-DDE

**DOI:** 10.1038/s41598-019-54579-z

**Published:** 2019-12-02

**Authors:** Nela Pavlíková, Petr Daniel, Jan Šrámek, Michael Jelínek, Veronika Šrámková, Vlasta Němcová, Kamila Balušíková, Petr Halada, Jan Kovář

**Affiliations:** 1 Department of Biochemistry, Cell and Molecular Biology & Center for Research of Diabetes, Metabolism, and Nutrition, Third Faculty of Medicine, Charles University, Prague, Czech Republic; 2 Department of Pathophysiology & Center for Research of Diabetes, Metabolism, and Nutrition, Third Faculty of Medicine, Charles University, Prague, Czech Republic; 3 Laboratory of Molecular Structure Characterization, Institute of Microbiology of the Czech Academy of Sciences, Prague, Czech Republic

**Keywords:** Cell signalling, Environmental sciences, Molecular medicine

## Abstract

Persistent organochlorine pollutants (POPs) gradually accumulate in the human organism due to their presence in the environment. Some studies have described a correlation between the level of POPs in the human body and the incidence of diabetes, but we know little about the direct effect of POPs on pancreatic beta-cells. We exposed pancreatic beta-cells INS1E to non-lethal concentrations of *p,p*′-DDT **(1,1′-(2,2,2-Trichloroethane-1,1-diyl)bis(4-chlorobenzene))** and *p*,*p*′-DDE (1,1′-(2,2-dichloroethene-1,1-diyl)bis(4-chlorobenzene)) for 1 month, and assessed changes in protein expression and the intracellular insulin level. 2-D electrophoresis revealed 6 proteins with changed expression in cells exposed to *p,p*′-DDT or *p,p*′-DDE. One of the detected proteins – vitamin D-binding protein (VDBP) – was upregulated in both cells exposed to *p,p*′-DDT, and cells exposed to *p,p*′-DDE. Both exposures to pollutants reduced the intracellular level of insulin mRNA, proinsulin, and insulin monomer; *p,p*′-DDT also slightly reduced the level of hexameric insulin. Overexpression of VDBP caused by the stable transfection of beta-cells with the gene for VDBP decreased both the proinsulin and hexameric insulin level in beta-cells similarly to the reduction detected in cells exposed to *p,p*′-DDT. Our data suggest that in the cells exposed to *p,p*′-DDT and *p,p*′-DDE, the increased VDBP protein level decreased the proinsulin expression in an unknown mechanism.

## Introduction

The fact that we are exposed to pollutants on a daily basis is a price we all pay for living in the modern world. The development in the chemical industry has allowed synthesizing compounds that have never existed in the environment before. Unfortunately, it also means that nature has limited options on how to degrade such compounds.

Persistent organochlorine pollutants (POPs) represent one of the major groups of contaminants in the environment and pose a severe threat to human health^1–3^. POPs’ chemical structures contain a covalent bond between carbon and chlorine that is not present in any natural compound^4,5^. As a consequence, in nature, no enzymes occur that can degrade the POPs^4,5^. Alternatives exist on how to degrade such compounds (e.g., photochemical reactions^6,7^, adapted bacteria), but they are extremely slow^8,9^. Therefore, POPs still contaminate soil^10,11^, air^12^, water^13,14^, and food^15^, but also human fat^16^, blood serum^17^, and breast milk^16,18,19^ even decades after most of the countries banned their use. Many POPs interfere with hormone signaling^20–22^, and this is why they are called endocrine disruptors.

DDT (1,1′-(2,2,2-Trichloroethane-1,1-diyl)bis(4-chlorobenzene)) was one of the most used pesticides in the world after World War II^23^. However, in the seventies, most of the countries banned its use due to its toxicity^24^. Currently, in humans, a large percentage of DDT has already been transformed into its metabolite DDE (1,1′-(2,2-dichloroethene-1,1-diyl)bis(4-chlorobenzene))^25,26^, but DDT still prevails in samples from the environment^25,27^. Moreover, some countries still use DDT as a pesticide in the fight against malaria^28^. Therefore, we are still far from being able to exclude DDT from the list of problematic organochlorine pollutants.

Some studies have described an association between the presence of certain POPs - including DDT and DDE - in the human body and the incidence of type 2 diabetes mellitus^26,29,30^. In type 2 diabetes mellitus, the failed signaling of insulin in target tissues (e.g., liver, muscles, fat tissue) called insulin resistance usually initiates the disease^31^ (unlike type 1 diabetes mellitus, which is an autoimmune disease where pancreatic beta cells gradually die, destroyed by the person’s own immune system^32^). The lack of insulin signaling is seen as a lack of insulin itself, and the organism demands more and more insulin from pancreatic beta cells^31^. Pancreatic beta cells gradually exhaust their ability to synthesize insulin and develop so-called stress of endoplasmic reticulum (ER stress) due to a growing amount of improperly folded proinsulin molecules (an inevitable side-effect of proinsulin synthesis) within the endoplasmic reticulum^33^. As a result, pancreatic beta cells stop synthesizing insulin in an attempt to get the situation under control. ER stress can result in the full recovery of pancreatic beta cells or their apoptosis^34^.

Exposure to POPs can affect two main processes involved in glucose homeostasis: secretion of insulin by pancreatic beta cells and insulin signaling in target tissues. Some studies found a connection between the presence of POPs in the human organism and insulin resistance^35–37^, suggesting that insulin signaling in target tissues can be the primary mechanism affected by POPs exposure. Another study revealed a negative effect of DDT on insulin secretion^38^ indicating that pancreatic beta cells can be the primary target. Nevertheless, we know little about the mechanism of any of these effects.

In the present study, we employed prolonged exposure to non-lethal doses of *p,p*′-DDT and *p,p*′-DDE - a model established in our previous work^39^ - to simulate the chronic effects of these compounds on pancreatic beta-cells. We examined how such exposure affected protein expression and insulin production in pancreatic beta-cells.

## Results

### Changes in protein expression analyzed by 2-dimensional electrophoresis

We used IPG (immobilized pH gradient) strips with a pH range of 4–7 to determine changes in protein expression after cell exposure to *p,p*′-DDT and *p,p*′-DDE. We also tested IPG strips with a pH range of 6–11, but the results were poor (data not shown).

**Figure 1:**
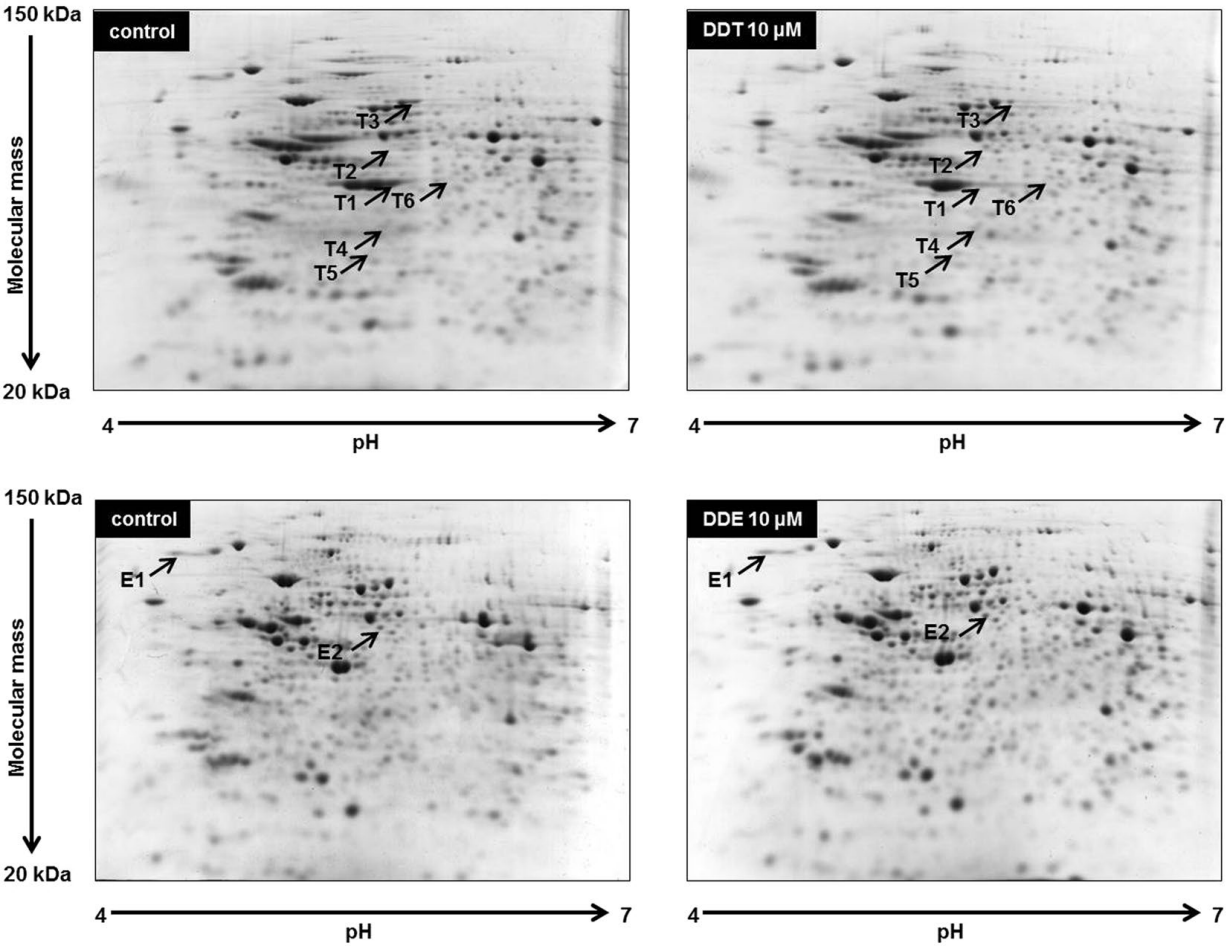
Representative 2-DE gels (pI range 4–7) of INS1E cells exposed to 10 μM *p,p*′-DDT or 10 μM *p,p*′-DDE for 1 month and cells exposed to solvent control (DMSO). (For pictures of all 2-D gels, see Supplementary data). The figure shows a set of representative 2-DE gels chosen from 3 independent sets of gels analyzing samples from 3 different experiments. Arrows mark spots representing proteins with the changed expression. Spot T1 represents actin; spot T2 represents vitamin D-binding protein; spot T3 represents mortalin/GRP75; spot T4 and spot T6 represent tubulin beta-5 chain; spot T5 represents annexin A4; spot E1 represents glucosidase 2 subunit beta precursor; spot E2 represents vitamin D-binding protein.

**Figure 2:**
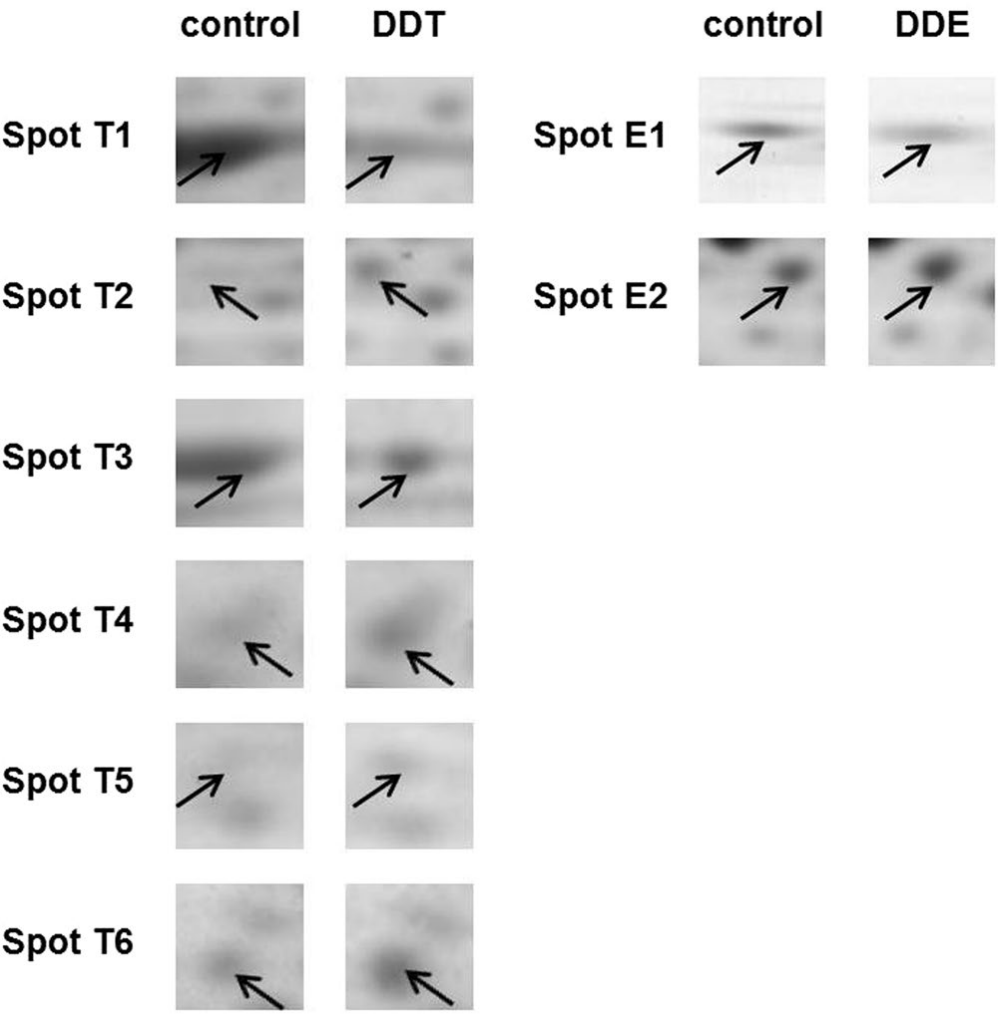
Details of spots with the changed expression on 2-DE gels (pI 4–7) of INS1E cells exposed to 10 μM *p,p*′-DDT, 10 μM *p,p*′-DDE, or solvent (DMSO) control for 1 month. Spot T1 represents actin; spot T2 represents vitamin D-binding protein; spot T3 represents mortalin//GRP75; spot T4 and spot T6 represent tubulin beta-5 chain; spot T5 represents annexin A4; spot E1 represents Glu2B (glucosidase 2 subunit beta precursor); spot E2 represents vitamin D-binding protein. For each spot, we took detailed pictures of corresponding spots from the pair of gels (one of three independent sets of gels analyzing samples from different experiments), which showed the most significant difference between the spot on control gel and DDT gel.

**Figure 3:**
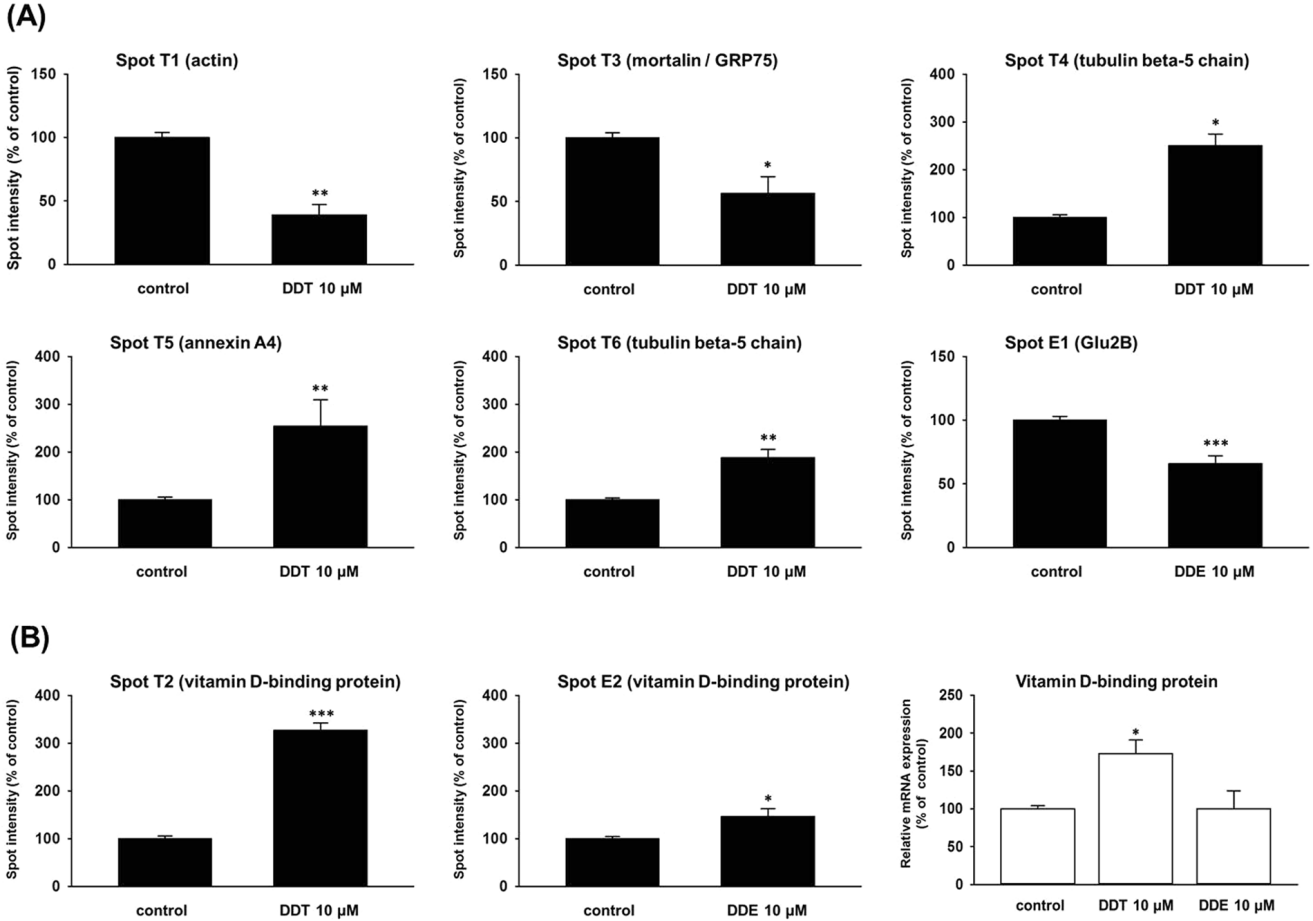
(**A**) Changes in protein expression found in INS1E cells exposed to p,p′-DDT (spot T1, T3-T6) or p,p′-DDE (spot E1) for 1 month when compared with the control. Columns represent mean values ± SEM of the intensity of matching spots from three independent sets of gels. Each set of gels represents samples from a different experiment. (**B**) Changes in protein expression of spots identified as vitamin D-binding protein (spot T2 and E2) and mRNA level of vitamin D-binding protein gene found in INS1E cells exposed to p,p′-DDT or p,p′-DDE for 1 month when compared with the control. For protein expression, columns represent mean values ± SEM of the intensity of matching spots from three independent sets of gels. Each set of gels represents samples from a different experiment. For the mRNA level, each column represents the mean of 4 experimental values ± SEM using samples from 2 different experiments. *Means a statistically significant difference when compared with the control (p < 0.05); **means a statistically significant difference when compared with the control (p < 0.01); ***means a statistically significant difference when compared with the control (p < 0.001). Mortalin/GRP75 is 75 kDa glucose-regulated protein, Glu2B is glucosidase 2 subunit beta precursor.

Approximately 450 spots were detected on each gel. (Fig. 1). We evaluated spots with the intensity altered by approximately 2-fold or more when compared with the control as spots with a changed expression. Exposure to *p,p*′-DDT changed the expression of six spots (see Fig. 1 and, for more details, Fig. 2). Mass spectrometry identified these spots as:(1) actin (spot T1) with expression decreased to 39% of the control, (2) mortalin/GRP75 (75 kDa glucose-regulated protein; spot T3) with expression decreased to 56% of the control, (3) tubulin beta-5 chain (spot T4 and spot T6) with expression increased to 251% and 188% of the control, (4) annexin A4 (spot T5) with expression increased to 254% of the control, and (5) vitamin D-binding protein (VDBP, spot T2) with expression increased to 327% of the control (Fig. 3, Table 1). We determined spot T4 as a protein fragment because its position on the gel differed from the predicted molecular size.

**Table 1:** Differentially expressed proteins found in INS1E cells exposed to p,p′-DDT (spot T1-T6) or p,p′-DDE (spot E1-E2) for 1 month when compared with the control identified by 2-DE electrophoresis and mass spectrometry.

Spot No.	Fold change	Protein name	DTB No.	No. peptides	Coverage [%]	MS/MS confirmation	MW protein	pI
T1	0.39	Actin, cytoplasmic 1 ↓	ACTB_RAT	10	29	AVFPSIVGRPR SYELPDGQVITIGNER	42	5.3
T2	3.27	Vitamin D-binding protein ↑	VTDB_RAT	6	15	SLSLILYSR RTQVPEVFLSK	54	5.7
T3	0.56	Mortalin/GRP75↓	GRP75_RAT	8	16	NAVITVPAYFNDSQR LLGQFTLIGIPPAPR	74	6.0
T4	2.51	Tubulin beta-5 chain ↑	TBB5_RAT	11	20	IREEYPDR FPGQLNADLR	50	4.9
T5	2.54	Annexin A4 ↑	ANXA4_RAT	10	34	NKPAYFAER GLGTDDSTLIR AASGFNATEDAQVLR	36	5.3
T6	1.88	Tubulin beta-5 chain ↑	TBB5_RAT	11	20	No	50	4.9
E1	0.66	Glucosidase 2 subunit beta precursor ↓	157818781	8	19	YEQGTGCWQGPNR SLEDQVETLR EVPPPQQPLRPPSPAEDEK	58	4.4
E2	1.49	Vitamin D-binding protein ↑	VTDB_RAT	8	24	SLSLILYSR RTQVPEVFLSK VPTANLEDVLPLAEDLTEILSR	54	5.7

The table includes the spot number, fold change, protein name, database number, the number of peptides matched to the identified protein, sequence coverage, peptide sequences confirmed by MS/MS, the theoretical molecular weight and isoelectric point (pI) of protein.

Exposure to *p,p*′-DDE failed to alter any spots complying with our 2-fold limit. Nevertheless, 2-D gels showed two spots with a significantly changed expression where the change was smaller than the 2-fold limit (see Fig. 1 and, for more details, Fig. 2). Mass spectrometry identified them as a glucosidase 2 subunit beta precursor (spot E1) downregulated to 66% of the control and vitamin D-binding protein (VDBP, spot E2) upregulated to 149% of the control (Fig. 3B, Table 1). That means that both exposures to pollutants increased the protein expression of VDBP in pancreatic beta cells.

Employing qRT-PCR, we determined the relative mRNA expression of the VDBP gene in pancreatic beta cells exposed to to p,p′-DDT or p,p′-DDE. The exposure to p,p′-DDT significantly increased the mRNA level of the VDBP gene to 173% of the control (Fig. 3B) while the exposure to p,p′-DDE did not significantly affect the mRNA level of the VDBP gene (Fig. 3B).

### Intracellular insulin level

**Figure 4:**
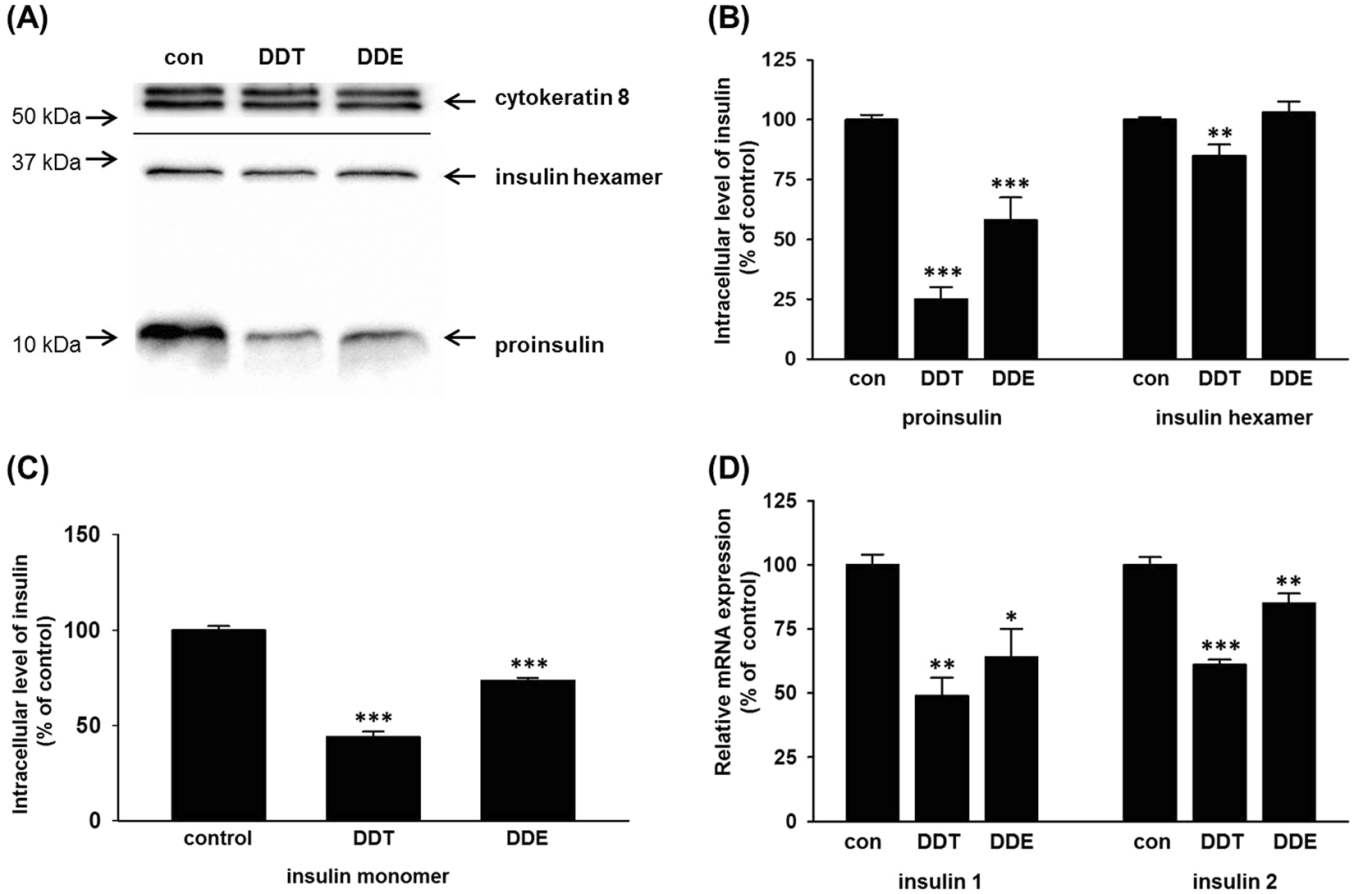
The intracellular level of insulin in INS1E cells exposed to 10 μM p,p′-DDT or 10 μM p,p′-DDE for 1 month compared with solvent (DMSO) control (con). (**A**) The figure shows a representative western blot. We used cytokeratin 8 as a loading control. We detected insulin hexamer and proinsulin at the same time at the same piece of membrane. (**B**) The graph represents the densitometry of western blots. Each column represents the mean of 8 experimental values ± SEM using samples from 4 different experiments. (**C**) The graph shows the insulin monomer level detected by the ELISA kit. Each column represents the mean of 9 experimental values ± SEM using samples from 4 different experiments. (**D**) The graph shows the relative mRNA expression of insulin 1 and insulin 2 genes. Each column represents the mean of 4 experimental values ± SEM using samples from 2 different experiments. *Means a statistically significant difference when compared with the control (p < 0.05); **means a statistically significant difference when compared with the control (p < 0.01); ***means a statistically significant difference when compared with the control (p < 0.001).

Western blot analysis revealed two bands related to insulin. One band, slightly below 37 kDa, represented hexameric insulin. The second band, slightly above 10 kDa, corresponded to proinsulin (81 amino acids, predicted size 10.5 kDa) (Fig. 4A). A 1-month exposure to *p,p*′-DDT reduced the intracellular level of both proinsulin and hexameric insulin in pancreatic beta cells INS1E. The expression of proinsulin decreased to 25% of the control, and the level of hexameric insulin to 85% of the control. In cells exposed to *p,p*′-DDE for 1 month, the expression of proinsulin decreased significantly to 58%; nevertheless, the level of hexameric insulin remained unchanged (Fig. 4B).

We used a commercial ELISA kit to determine the intracellular level of the insulin monomer because the insulin monomer is too small (5 kDa) to be detected by a western blot. In pancreatic beta cells INS1E, a 1-month exposure to p,p′-DDT reduced the intracellular level of the insulin monomer to 45% of the control. A 1-month exposure to p,p′-DDE reduced the intracellular level of the insulin monomer to 73% of the control (Fig. 4C).

Employing qRT-PCR, we determined the relative mRNA expression of rat insulin 1 and insulin 2 genes. The exposure to p,p′-DDT significantly decreased the mRNA level of insulin 1 gene to 49% of the control, and insulin 2 gene to 61% of the control (Fig. 4D). The exposure to p,p′-DDE significantly decreased the mRNA level of insulin 1 gene to 64% of the control, and insulin 2 gene to 85% of the control (Fig. 4D).

### Effect of VDBP overexpression

**Figure 5:**
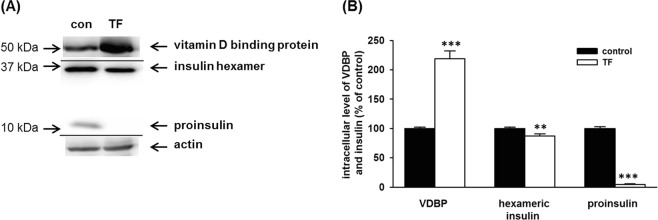
Effect of VDBP overexpression on insulin level in INS1E cells. (**A**) The figure shows a representative western blot (actin represents a loading control). Transfected cells (TF) were stably transfected with the VDBP gene; cells of control (con) were transfected with plasmid without VDBP (vitamin D-binding protein) gene. Insulin hexamer and proinsulin were detected at the same time at the same piece of membrane. (**B**) The graph represents the densitometry of western blots. Each column represents the mean of 8 experimental values ± SEM using samples from 4 different experiments. **Means a statistically significant difference when compared with the control (p < 0.01); ***means a statistically significant difference when compared with the control (p < 0.001).

In cells stably transfected with the VDBP gene, we determined the intracellular insulin level using western blot analysis (Fig. 5). We used INS1E cells transfected with a plasmid lacking the VDBG gene as a control. Transfection significantly increased the intracellular level of VDBP (219% of the control) and decreased the expression of proinsulin (6% of the control). It also decreased the level of hexameric insulin (87% of the control) (Fig. 5).

## Discussion

In the present study, we tested the effects of the organochlorine pollutants - *p,p*′-DDT and *p,p*′-DDE - on the protein expression and intracellular insulin production in pancreatic beta-cells. The *p,p*′-DDT isomer represents the major component of the DDT mixture used as pesticide^40^, and *p,p*′-DDE is its corresponding metabolite^41^. To examine the impact of pollutants stored in human tissues on human health, researchers usually employ epidemiological studies^16,19,26^. No one has ever exposed a glucose-responsive pancreatic beta-cell line to *p,p*′-DDT and *p,p*′-DDE for a longer time than a few days to examine the effect of such conditions on protein expression and intracellular insulin production.

For a 1-month exposure, we needed a concentration high enough to induce changes in protein expression detectable by 2-D electrophoresis, and low enough to allow beta-cells to survive a 1-month exposure in good condition. From our previous experiment^39^, we knew that lower and more environmentally relevant concentrations^16,19,26^ of *p,p*′-DDT and *p,p*′-DDE (0.1 μM and 1 μM) showed no effect detectable by 2-D electrophoresis after a 1-month exposure. For that reason, we chose a 10 μM concentration of both *p,p*′-DDT and *p,p*′-DDE for our experiment.

2-D electrophoresis revealed 6 proteins with changed expression in cells exposed to *p,p*′-DDT or *p,p*′-DDE. We reviewed the literature to evaluate the possible impact of the altered expression of detected proteins on pancreatic beta cells’ function.

The exposure to *p,p*′-DDT increased the level of annexin A4 in pancreatic beta-cells^42^. Annexin A4 binds calcium ions^43^ and decreases the level of cAMP within the cells^44^. Both calcium ions and cAMP are essential members of the signaling pathway leading to exocytosis of insulin vesicles^45,46^. Therefore, the overexpression of annexin A4 can play a role in insulin secretion.

Mortalin/GRP75, reduced in cells exposed to *p,p*′-DDT, protects the mitochondria against oxidative stress^47–49^. According to Burbulla and coworkers^48^, the downregulation of mortalin/GRP75 represents a severe threat for mitochondria and can ultimately lead to a decrease of total mitochondrial mass in the cell, and reduced synthesis of ATP. Pancreatic beta cells need an increased level of ATP to close the K^+^ channels and induce membrane depolarization, which is a part of the signaling pathway that leads to insulin secretion^46^. Hence, decreased mortalin/GRP75 expression in cells exposed to *p,p*′-DDT could signal problems with mitochondria and with signaling leading to insulin secretion.

Exposure to *p,p*′-DDT downregulated the expression of actin and upregulated the expression of the tubulin beta-5 chain. Microtubules serve as a highway for the transport of insulin vesicles to the cytoplasmic membrane^46^. Before reaching the membrane, insulin vesicles have to make their way through the actin cortex that mechanically supports the membrane^45,50^. Therefore, the altered expression of proteins that compose microtubules or actin cortex can impact insulin secretion. We have detected an altered expression of some cytoskeletal proteins and their fragments in our previous work using a human beta cell-line^39^ and discussed their possible meanings there.

Exposure to *p,p*′-DDE decreased expression of a glucosidase 2 subunit beta precursor in INS1E pancreatic beta cells. This protein represents a part of the enzyme that glycosylates proteins in the endoplasmic reticulum^51^. Its downregulation could indicate some problems in the endoplasmic reticulum, which is the organelle where the insulin is processed.

Both exposure to *p,p*′-DDT and exposure to *p,p*′-DDE increased expression of the vitamin D-binding protein (VDBP), a protein that serves as a transporter for vitamin D in the blood^52^. Vitamin D is a hydrophobic compound^53^ just like *p,p*′-DDT and *p,p*′-DDE. A possibility exists that VDBP can also bind *p,p*′-DDT and *p,p*′-DDE. In such a case, VDBP upregulation would decrease the bioavailability of *p,p*′-DDT and *p,p*′-DDE within pancreatic beta-cells^52^.

Several authors^54–56^ described a correlation between variations in the VDBP gene and the presence of antibodies against VDBP in the blood and the incidence of several types of diabetes (i.e., type 1 diabetes mellitus, type 2 diabetes mellitus, and gestational diabetes)^57–59^. However, those studies examined the level of VDBP in the blood or urine^57–59^ and not in pancreatic beta-cells.

We wanted to check if the exposure to tested compounds affected the intracellular level of insulin. Western blot revealed two types of insulin: proinsulin (a precursor of insulin) and hexameric insulin (a final form of insulin ready for secretion)^46^. Employing ELISA assay, we detected insulin monomer, an active form of insulin. Normally, only hexameric insulin and no insulin monomers occur in insulin vesicles in pancreatic beta cells^60,61^. Hexameric insulin dissolves into insulin monomers after reaching the extracellular fluid and blood^60,61^. In our samples, the process of lysing the cells probably caused the dissolving of some hexameric insulin into insulin monomers.

The exposure to pollutants decreased the level of an insulin precursor (proinsulin) substantially (see Fig. 4), but the level of the final form of insulin ready for export (hexameric insulin) dropped only slightly (but significantly) in the cells exposed to p,p′-DDT and not at all in the cells exposed to p,p′-DDT. We hypothesize that the 1-month exposure to pollutants decreased the proinsulin level gradually. The relative lack of proinsulin starts to play a role only when the cell needs to replenish a depleted number of insulin vesicles. A more severe shortage of proinsulin will affect the level of hexameric insulin sooner. We hypothesize that after 1-month exposure, the lack of proinsulin in cells exposed to p,p′-DDT was severe enough to influence the level of hexameric insulin. In cells exposed to p,p′-DDE, the downregulation of proinsulin was less significant than in cells exposed to p,p′-DDT and the level of hexameric insulin remained unchanged.

The exposure to both pollutants decreased the level of the insulin monomer more than the level of hexameric insulin, which is a source of insulin monomer. We do not know how, when, and why the hexameric insulin dissolves in cell lysates and we cannot exclude cross-reactivity between hexameric insulin and insulin monomer so we cannot correctly evaluate these results.

Quantitative RT-PCR revealed a decreased mRNA level of both rat insulin 1 and 2 genes after exposure to p,p′-DDT and p,p′-DDE. Nevertheless, in the case of p,p′-DDT, the insulin mRNA did not decrease enough to explain the low level of proinsulin. We hypothesize that the exposure to p,p′-DDT and p,p′-DDE also affected the proinsulin expression in another way than just by decreasing the transcription of insulin genes (e.g., by influencing the translation of insulin mRNA).

We can conclude that both pollutants negatively affected insulin production in pancreatic beta cells INS1E; p,p′-DDT more than p,p′-DDE. The reason why the molecule of p,p′-DDT affected pancreatic beta cells more than a molecule of p,p′-DDE could be better internal mobility of the molecule of p,p′-DDT. The molecule of p,p′-DDE has one more double bond than the molecule of p,p′-DDT. A double bond does not allow rotation along its axis and limits the number of conformations the molecule can take. That also limits its ability to fit into various reactive places on different molecules.

From all proteins detected by 2-D electrophoresis, vitamin D-binding protein (VDBP) was the only one known to be somehow connected with the incidence of diabetes^55,57–59,62^. We wanted to examine whether the upregulation of VDBP expression played a role in the decrease of proinsulin and hexameric insulin levels in pancreatic beta-cells. Cells overexpressing VDBP showed a reduction of both the proinsulin and hexameric insulin level (Fig. 5) very similar to the downregulation found in cells exposed to *p,p*′-DDT (see Fig. 4). We used cells transfected with empty plasmid as a control, so the difference in the expression was not the result of the treatment during transfection. Our data suggest that the exposure to *p,p*′-DDT increased the protein level of VDBP in pancreatic beta-cells which subsequently decreased the level of proinsulin. The exposure to *p,p*′-DDE upregulated VDBP less than the exposure to *p,p*′-DDT, and thus the effect on proinsulin level was less significant.

Our data suggest that intracellular VDBP affects the level of proinsulin in pancreatic beta-cells. If this mechanism also exists in human pancreatic beta-cells, VDBP can play an essential role in further research concerning diabetes and its treatment^52,53^. Interestingly, Kuo and coworkers^63^ have recently published a study describing that VDBP-knock out mice preserved insulin secretion when fed with a high-fat diet. That supports our theory that VDBP can play an essential role in disturbing insulin production in pancreatic beta-cells.

Our data have produced many questions that we would like to answer: What kind of mechanism is involved in VDBP-mediated downregulation of proinsulin? How is the level of VDBP regulated within pancreatic beta-cells? Does any connection exists between VDBP in the blood (where it binds vitamin D) and VDBP within pancreatic beta-cells and is vitamin D involved? All these questions will be addressed in our further research.

## Conclusions

In this study, we have shown that a 1-month exposure to a non-lethal dose (10 μM) of *p,p*′-DDT increased the expression of the vitamin D-binding protein, tubulin beta-5 chain, and annexin A4; and decreased the expression of actin, and mortalin/GRP75 in rat pancreatic beta-cells INS1E. The 1-month exposure to a non-lethal dose (10 μM) of *p,p*′-DDE increased the expression of the vitamin D-binding protein and decreased the expression of a glucosidase 2 subunit beta precursor in rat pancreatic beta-cells INS1E. The exposure to *p,p*′-DDT significantly increased the mRNA level of the VDBP gene in rat pancreatic beta-cells INS1E. The exposure to *p,p*′-DDT decreased the intracellular level of proinsulin, hexameric insulin, and insulin monomer. The exposure to *p,p*′-DDE decreased the intracellular level of proinsulin, insulin monomer, but not hexameric insulin. Both exposure to *p,p*′-DDT and *p,p*′-DDE decreased the mRNA level of insulin 1 and insulin 2 genes. In the cells transfected with the gene for VDBP, the overexpression of VDBP resulted in the decreased intracellular level of proinsulin and hexameric insulin. Our results suggest that the overexpression of the vitamin D-binding protein (VDBP) played an essential role in the decrease of insulin production after exposure to *p,p*′-DDT, and also, to a lesser extent, after exposure to *p,p*′-DDE. Other detected proteins with the changed expression, e.g., annexin A4 or tubulin beta-5 chain, could also play a a certain role in the decrease of insulin production. We are the first who have described the connection between the intracellular level of VDBP and proinsulin expression by pancreatic beta-cells. This connection could represent a significant contribution to our understanding of the physiology of pancreatic beta-cells including the effect of some organochlorine pollutants.

## Material and Methods

### Material

We obtained *p,p*′-DDT (1,1,1-Trichloro-2,2-bis(4-chlorophenyl)ethane; 31041-100MG), *p,p*′-DDE (1,1-Dichloro-2,2-bis(4-chlorophenyl)ethane); 35487-100MG), and RPMI medium from Sigma-Aldrich (https://www.sigmaaldrich.com); rabbit polyclonal antibody against insulin (15848-1-AP), and rabbit polyclonal antibody against vitamin D-binding protein (16922-1-AP) from Proteintech (https://www.ptglab.com); rabbit polyclonal antibody against cytokeratin 8 (ab154301) from Abcam (http://www.abcam.com); mouse monoclonal antibody against actin (ab11003), and mouse monoclonal ANTI-FLAG® M2 (F1804) antibody from Sigma-Aldrich (https://www.sigmaaldrich.com). We obtained Gene Expression Assay Hprt1 (Rn01527840_m1), Gc (Rn00561256_m1), rat insulin 1 (Rn01774648_g1), and rat insulin 2 (Rn02121433_g1) from Life Technologies (https://www.thermofisher.com › *home* › *brands* › *life-technologies*).

### Cell culture

Rat pancreatic beta-cells INS1E with glucose-inducible insulin secretion were routinely cultured in a medium based on RPMI 1640, which contained phenol red, sodium pyruvate (110 μg/ml), extra L-glutamine (300 μg/ml), HEPES (15 mM), streptomycin (100 μg/ml), and penicillin (100 U/ml) as previously described^64–66^. We passaged the cells once per week and replaced the medium with a fresh one 4 days after the passage. The medium was also supplemented with 10% fetal bovine serum (FBS). Cells were routinely maintained in a humidified atmosphere of 5% CO_2_, in the air, at 37 °C^67^.

### Exposure to p,p′-DDT and p,p′-DDE

For the experiment, we maintained INS1E cells as described above for 1 month in the medium described above, which contained *p,p*′-DDT or *p,p*′-DDE (10 μM), or DMSO as the solvent control. The concentration of DMSO in the medium was 0.5%. After 4 weeks of exposure, we harvested the cells.

### 2-D Electrophoresis

We trypsinized the cells, washed them 3-times with ice-cold PBS, and resuspended them in Protein Extraction Buffer-V (GE Healthcare, http://www.gelifesciences.com) (urea, thiourea, CHAPS) containing a 2% Protease Inhibitor Mix (GE Healthcare, http://www.gelifesciences.com). We purified all samples using a 2-D Clean-Up Kit (GE Healthcare, http://www.gelifesciences.com) following the manufacturer´s instructions. We determined protein concentrations using a 2-D Quant Kit (GE Healthcare, http://www.gelifesciences.com), which was compatible with components of the Protein Extraction Buffer-V.

We performed the isoelectric focusing, equilibration, the second dimension, and the staining of gels as previously^39^. The MALDI Mass Spectrometry and Protein identification were performed following the protocol described previously^68^.

### Cloning

We extracted the total RNA from the INS1E rat cell line using an RNeasy Mini Kit (Qiagen, https://www.qiagen.com/cn/products/). We synthesized the first strand of cDNA (Maxima H Minus First Strand cDNA Synthesis Kit (Thermo Fisher Scientific, https://www.thermofisher.com)) using a modified manufacturer´s protocol with oligo(dT)18 primer (polymerase reaction was carried out at 50 °C for 30 minutes followed by 55 °C for 30 minutes). We used the end product as a template for two-rounds of polymerase chain reaction (PCR). The entire vitamin D binding protein ORF (VDB) without stop codon was synthesized with the following primers: 5′-CCGCCACCATGAAGAGGGTTCTGGTTCTCC-3′ (VDB KpnI forward primer 1) and 5′-GCTAGCGGACTGCAGGATGTCTCTCATTTC-3′ (VDB NheI reverse primer 1) for the first round (using Q5® High Fidelity Polymerase (NEB, https://www.neb.com/)). Then, we cleaned the PCR product (GenElute PCR Clean-up Kit, Sigma-Aldrich, https://www.sigmaaldrich.com) and reamplified it using primers: 5′-ATGCATAGGTACCGCCACCATGAAGAGGGTTC-3′ (VDB KpnI forward primer 2) and 5′-ATCAATCGCTAGCGGACTGCAGGATGTC-3′ (VDB NheI reverse primer 2) for the second round. The PCR product was digested with KpnI and NheI restriction endonucleases and we separated it using agarose gel electrophoresis. We excised the band corresponding to the length of VDB ORF and ligated it into the KpnI-NheI sites of the pcDNA3.1b-FLAGC plasmid, in the frame with the C-terminal FLAG, to generate the pcDNA3.1b–Ra-VDB-FLAGC expression construct. We checked the insert by enzyme digestion as well as by sequencing (GATC Biotech, https://www.eurofinsgenomics.eu).

We prepared the pcDNA3.1b-FLAGC plasmid by replacing the neomycin resistance gene in the original pcDNA3.1 with the blasticidin resistance gene from pcDNATM6.2-DEST via the XmaI-BsmI sites (Thermo Fisher Scientific, https://www.thermofisher.com). The sequence coding C-terminal FLAG (fused with the NheI restriction site) and two stop codons (-ASDYKDDDDK**) were ligated as an oligonucleotide into the XhoI site.

### Stable Transfection with Gene for Vitamin D-binding Protein

For stable transfection, we transfected INS1E cells with pcDNA3.1b–Ra-VDB-FLAGC or pcDNA3.1b-FLAGC (mock) in 6-well plates for 24 hours. We seeded the cells at a 1:5 ratio into 10 cm Petri dishes and cultivated them with 8 µg/ml blasticidin (InvivoGen, https://www.invivogen.com/) for three weeks. We picked the colonies using cloning cylinders (Sigma-Aldrich, https://www.sigmaaldrich.com) and transferred them into a growth medium containing 2 µg/ml blasticidin. One of five clones stably expressed the VDB-FLAG protein and was chosen for further assays as well as the clone that was resistant to blasticidin (mock).

### Western blot

We performed western blot as described previously^64,69^ with minor modifications. We used 10 μg samples of total protein (whole cell lysates) for separation; we used 18% polyacrylamide gel for analysis of insulin and 10% polyacrylamide gel for analysis of the vitamin D-binding protein level. We applied the following dilutions of primary antibodies: 1:3000 for rabbit polyclonal antibody against insulin (15848-1-AP), 1:1500 for rabbit polyclonal antibody against vitamin D-binding protein (16922-1-AP), 1:1000 mouse monoclonal antibody against actin (ab11003), and 1:1000 for rabbit polyclonal antibody against cytokeratin 8 (ab154301). We analyzed the optical density of bands using Image Master™ 2D Platinum 6.0 software (GE Healthcare, Uppsala, Sweden).

### Elisa

We analyzed the level of intracellular insulin using the Mercodia Rat Insulin ELISA kit (https://www.mercodia.se/, 10-1250-01). We used samples from four independent experiments for the ELISA experiment. We diluted our samples to the concentration 1 μg/μl and diluted them 1:5000 with the Mercodia Diabetes Sample Buffer (https://www.mercodia.se/, 10-1195-01) for the experiment. We performed the ELISA experiment following the manufacturer´s instructions. After the ELISA experiment, we determined the protein concentration of the 1 μg/μl sample solutions using the BCA kit (Pierce^TM^ BCA Protein Assay Kit, #23227) and adjusted the results from ELISA to the real concentrations of the sample solutions.

### RNA isolation and qRT-PCR

We isolated total RNA using the RNeasy Mini Kit (Qiagen, Hilden, Germany) with on-column DNA digestion according to the manufacturer’s recommendations. We measured the concentration of isolated RNA with Implen NanoPhotometerTM (Implen, Munich, Germany). 1 µg of total RNA was reverse-transcribed into cDNA using a Maxima H minus Reverse Transcriptase kit (Thermo Fisher Scientific, Waltham, MA, USA). We used the equivalent of 20 ng for RT-qPCR on ABI-PRISM 7500. Each reaction was run in triplicates using TaqMan® gene expression assays (Hprt1: Rn01527840_m1; INS1: Rn02121433_g1; INS2: Rn01774648_g1 and GC: Rn00561256) andTaqMan® Fast Advanced Master Mix (Thermofisher Scientific, USA). We normalized gene expression of target genes to the expression of Hprt1. We calculated the fold change of expression using the ΔΔCt method.

### Statistical analysis

We analyzed the significance of differences between matching spots (2-D gels) and bands (western blot) using the Student’s t-test. We analyzed the significance of differences in intracellular insulin ELISA assay and RT-PCR using a one-way ANOVA Dunnett’s test (SigmaPlot Software 14.0).

## Supplementary information

Supplementary data

## Data Availability

The datasets generated and/or analyzed during the current study are available from the corresponding author on reasonable request.
