# Supplementary material for: Upregulation of vitamin D-binding protein is associated with changes in insulin production in pancreatic beta-cells exposed to p,p′-DDT and p,p′-DDE
Source: Sci Rep. 2019 Dec 2;9:18026. doi: 10.1038/s41598-019-54579-z (PMC6889289; doi:10.1038/s41598-019-54579-z)

Upregulation of vitamin D-binding protein is associated with changes in insulin production in pancreatic beta-cells exposed to p,p'-DDT and p,p'-DDE

*Nela Pavlikova[1], Petr Daniel[1], Jan Sramek[1], Michael Jelinek[1], Veronika Srámkova[2], Vlasta Nemcova[1], Kamila Balusikova[1], Petr Halada[3], Jan Kovar[1]

[1] Department of Biochemistry, Cell and Molecular Biology & Center for Research of Diabetes, Metabolism, and Nutrition, Third Faculty of Medicine, Charles University, Prague, Czech Republic

[2] Department of Pathophysiology & Center for Research of Diabetes, Metabolism, and Nutrition, Third Faculty of Medicine, Charles University, Prague, Czech Republic

[3] Laboratory of Molecular Structure Characterization, Institute of Microbiology of the Czech Academy of Sciences, Prague, Czech Republic

*Corresponding author:

Nela Pavlikova (nela.pavlikova@lf3.cuni.cz)

Phone: +420267102657, Fax: +420267102650

3LF UK, Dep. of Biochemistry, Cell and Molecular Biology

Ruska 87

100 00 Prague

Czech Republic

1. **Original western blots cropped in Figure 4**

All western blots were cropped (and horizontally flipped, if necessary) using Google Picasa. We did not use any software to increase the contrast. The low background is a result of using 5 % low-fat milk for blocking solution, first, and secondary antibody. All bands from one figure origin from the same gel/membrane that was horizontally cut and exposed to different primary antibodies. In some cases, the membrane was stripped, blocked and exposed to another primary antibody.

1.1. Hexameric insulin and proinsulin.

Blot used in figure 4. We detected hexameric insulin (upper bands) and proinsulin (lower bands) at the same membrane using the same primary antibody.

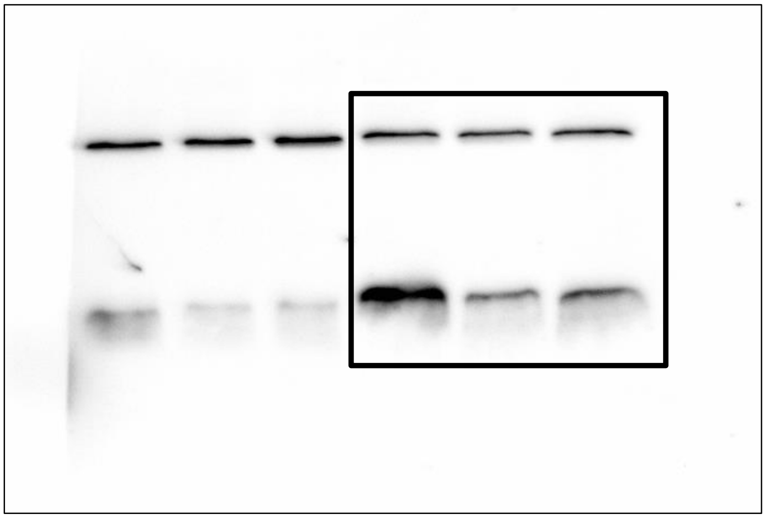

****

1.2. Loading control – cytokeratin 8.

Blot used in figure 4. The membrane was stripped before it was used for detecting cytokeratin 8. We used cytokeratin 8 because both actin and tubulin changed their expression in cells exposed to DDT. GAPDH has the same position as hexameric insulin. The artifacts are results of both stripping and using samples in the buffer for 2-DE (the buffer contains urea) which is not ideal for western blot.

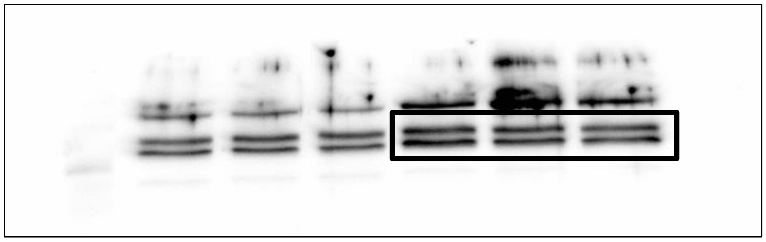

2. Original western blots cropped in Figure 5

All western blots were cropped (and horizontally flipped, if necessary) using Google Picasa. We did not use any programme to increase the contrast. The low background is a result of using 5 % low-fat milk for blocking, first, and secondary antibody. All bands from one figure origin from the same gel/membrane that was horizontally cut and exposed to different primary antibodies. In some cases, the membrane was stripped, blocked and exposed to another primary antibody.

2.1. Vitamin D-binding protein.

Blot used in figure 5. VDBP is the upper band with corresponding size 50 kDa. We used four different sets of lysates (control cells – transfected cells).

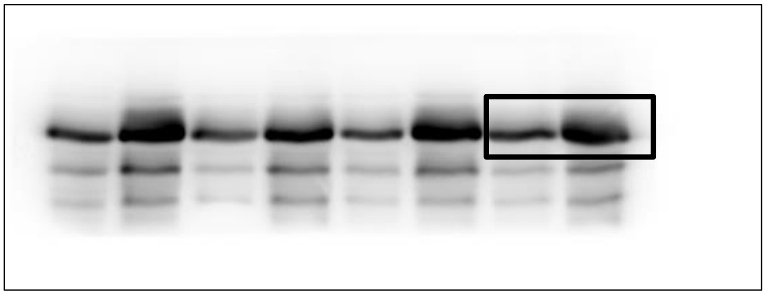

****

VDBP. Another western blot of lysates of transfected cells (control cells – transfected cells), from different transfection.

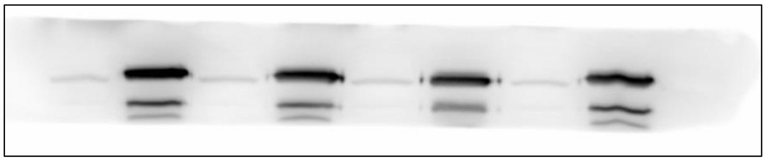

****

2.2. Hexameric insulin and insulin.

Blot used in figure 5. We used four different sets of lysates (control cells – transfected cells). For this western blot, we tested 10 % polyacrylamide gels instead of 18 % polyacrylamide gels. That is the reason why the distance between two lines of bands is shorter at the upper gel than at the lower gel.

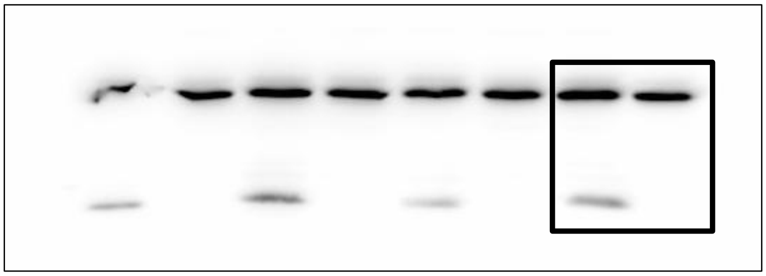

****

Hexameric insulin and insulin. This blot is one of the less successful blots run on 18 % polyacrylamide gel. We used four different sets of lysates (control cells – transfected cells). We do not know why the proinsulin created triple bands. It happened just once.

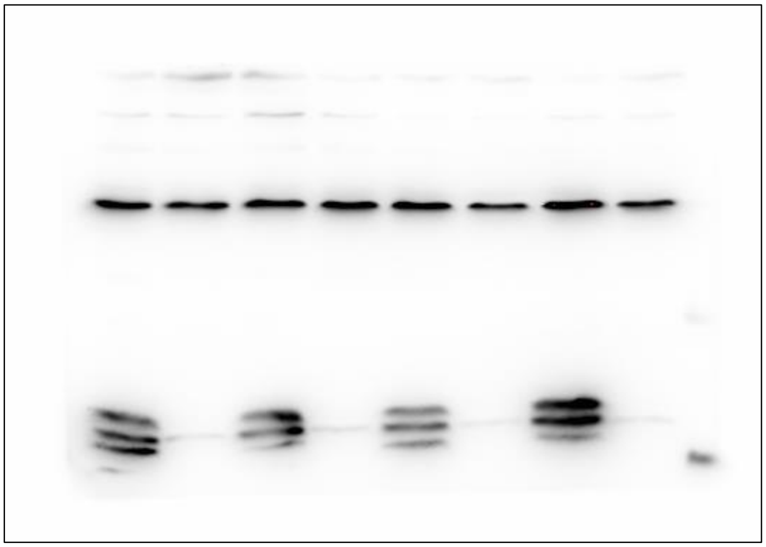

****

2.3. Loading control - actin.

Blot used in figure 5. We used actin as a loading control for western blots of transfected cells.

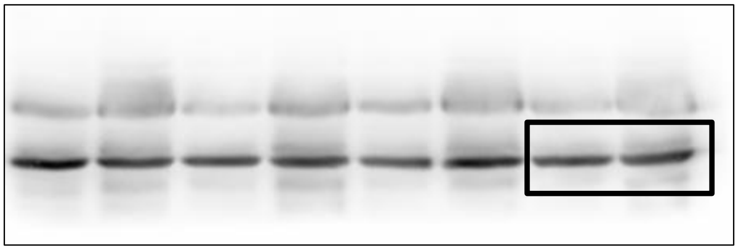

3. 2-DE gels – exposure to DDT

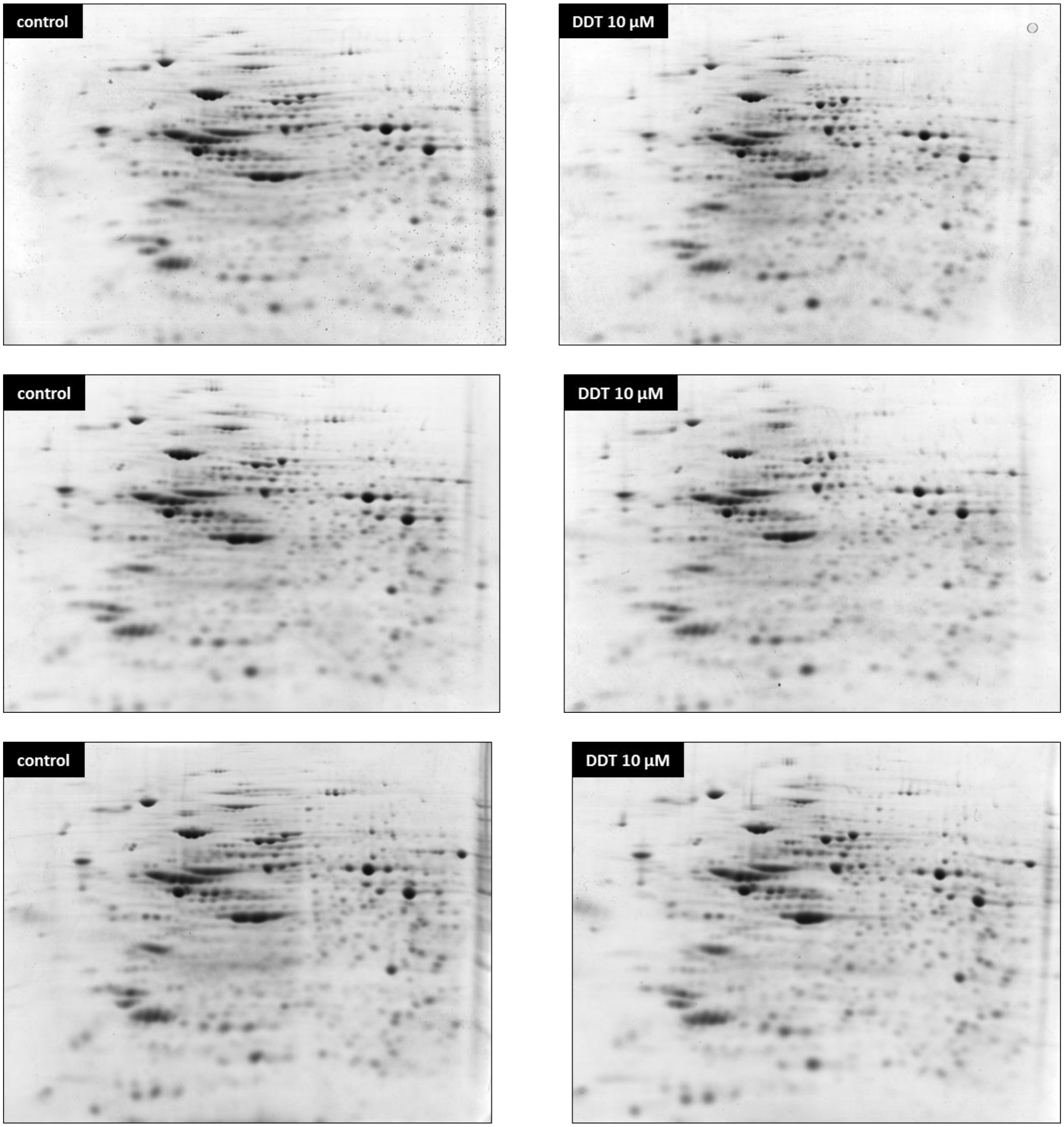

4. 2-DE gels – exposure to DDE

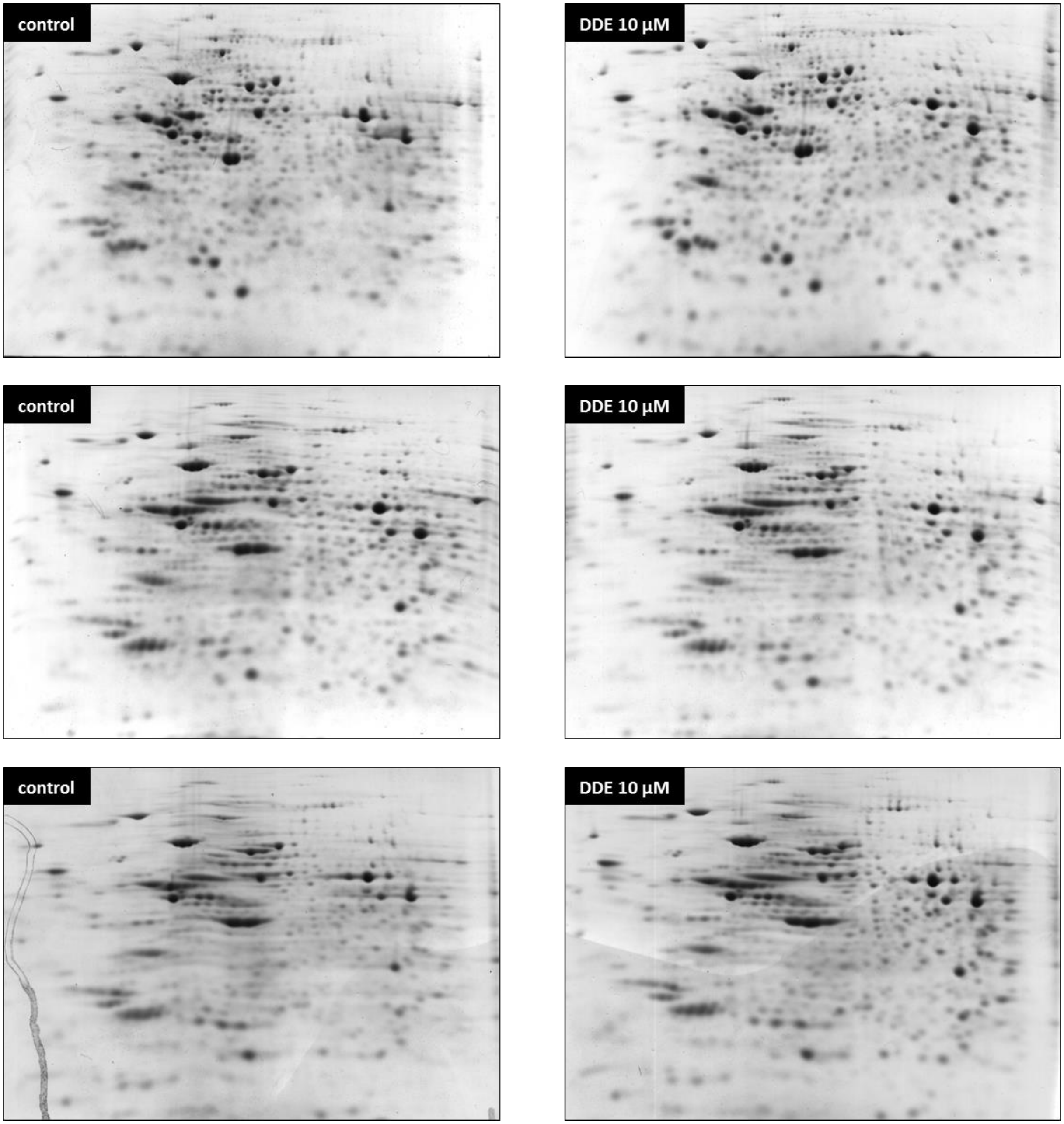

Supplement: Supplementary file 1 — Supplementary data [file 41598_2019_54579_MOESM1_ESM.pdf]
